# Polyphenols in Sugar Beet Leaves: Composition, Variability, and Valorization Opportunities

**DOI:** 10.3390/molecules31030489

**Published:** 2026-01-30

**Authors:** Aneta Antczak-Chrobot, Jakub Macierzyński, Maciej Wojtczak

**Affiliations:** 1 Department of Sugar Science and Food Safety Management, Faculty of Biotechnology and Food Sciences, Lodz University of Technology, Wólczańska 171/173, 90-530 Łódź, Poland; 2 Central Laboratory, Nofer Institute of Occupational Medicine, św. Teresy 8, 91-348 Łódź, Poland; jakub.macierzynski@imp.lodz.pl; 3 Department of Inorganic and Analytical Chemistry, Faculty of Chemistry, University of Lodz, Tamka 12, 91-403 Łódź, Poland; maciej.wojtczak@chemia.uni.lodz.pl

**Keywords:** petioles, blades, polyphenols, vitexin, proteins, HPLC-DAD, LC-MS

## Abstract

Sugar beet (root) is primarily used by industry as a raw material for sugar production, and its large-scale cultivation is closely linked to the sugar industry. Currently, sugar beet leaf (SBL) is not processed and is typically left on the field as green fertilizer after mechanical harvesting. This represents an underutilized biomass stream with potential bioactive compounds. The aim of this study was to evaluate the distribution of polyphenol and proteins in the leaf blade and petioles of different sugar beet cultivars harvested at various time points. Total polyphenols were quantified using vitexin as a reference standard, and the phenolic profile of methanolic extracts was characterized using complementary HPLC-DAD and LC-MS methods. The protein content in leaf blades ranged from 19% to 29% (dry weight) and was significantly influenced by cultivar and harvest date. Petioles contained significantly lower protein content, ranging from 4.9% to 9.5% (dry weight). The total polyphenol content (TPC) varied with cultivar and harvest time, ranging from 7.8 to 11.0 mg/g DW in leaf blades and from 0.8 to 2.7 mg/g DW in petioles. Leaf blades also contained substantially higher concentrations of vitexin derivatives (mean 7.4 ± 2.3 mg/g DW) than petioles (1.1 ± 0.6 mg/g DW). The percentage contribution of vitexin derivatives to TPC was high in both tissues (>70%) and decreased with later harvest dates. The results provide a detailed characterization of polyphenolic and protein distribution in blades and petioles of sugar beet leaves and can support further evaluation of their potential use in value-added applications.

## 1. Introduction

Sugar beet (*Beta vulgaris* L. subsp. *vulgaris*) is a cornerstone crop for the European sugar industry. Poland is a significant producer of sugar beets; according to Polish Sugar Association data, the production volume was over 16 million tons in 2023/2024 [1]. Sugar beet root is the main source of sugar production in temperate climates. Approximately 75% of the beet’s mass is water, and the main reserve substance, 15–20%, is sucrose. The remaining dry matter is mostly composed of building blocks: primarily pectin, pectin-forming compounds, and cellulose. Unlike the root, the aboveground part of the plant (the leaves) contains significantly higher levels of nutrients and bioactive compounds. This is because leaves are primarily responsible for metabolic and biosynthetic processes rather than storage. They contain antioxidant and antibacterial compounds including flavonols, saponins, quercetin, apigenin, folic acid, ferulic acid, omega-3 and omega-6 fatty acids, and others [2,3].

During mechanical harvesting, the above-ground biomass (SBL) is routinely cut and left on the field as green fertilizer. While this practice returns nutrients and organic matter to the soil, it also represents a substantial underutilization of a consistent, seasonal stream of plant biomass with provable potential of high-value compounds in circular “waste to resource” strategies [2,3,4]. In that context, SBL should be increasingly viewed not as a field residue but as a feedstock for integrated, low-footprint biorefineries positioned near the point of biomass generation [4].

Chemically, SBL comprises structural carbohydrates (cellulose, hemicelluloses, and pectin), proteins (including RUBISCO-rich fractions), lipids, minerals, and specialized metabolites [5]. Reported dry matter composition indicates substantial protein alongside carbohydrate and fiber fractions, with ranges shaped by cultivar, season, and agronomy [4,5]. In specialized metabolites, phenolic acids (e.g., caffeic/ferulic derivatives) and flavonoids (flavones) are prominent and can be easily resolved using modern LC-MS/MS methods [6,7]. Beyond phenolics, SBL contains typical triterpenoid saponins. These components are important for plant function and resistance, as well as their potential valorization [8,9].

In sugar beet leaves, both polyphenol content and biological activity change over the course of development; high values are often observed around day ~60 (younger leaves), although some methods and post-processing steps yield later maxima (e.g., higher polyphenol content after dehydration at day ~100) [3]. The relationship between total phenolic content and anti-inflammatory/antibacterial activity is generally positive, but depends on compound profile and assay conditions [6].

The techno-economic rationale for SBL valorization is reinforced by the accordance of leaf protein recovery with phenolic extraction. On the protein side, leaf protein concentrates obtained via mild thermal or pH coagulation and membrane steps show favorable amino-acid profiles and techno-functional properties (solubility, foaming, and emulsification), enabling co-products for food, feed, or materials [10,11]. On the phenolic side, scalable routes span aqueous/buffer extraction, enzyme-assisted disruption, and membrane separations; more recently, green-solvent concepts such as natural deep eutectic solvents (NADES) have been explored to improve selectivity and sustainability [12,13].

Despite this promise, industrial uptake of SBL remains limited. Key bottlenecks include (i) heterogeneity in biomass composition driven by genotype, environment, and harvest timing; (ii) co-extraction of chlorophylls, lipids, and nucleic acids that can impair color, flavor, and functionality; (iii) high endogenous polyphenol oxidase (PPO) activity that accelerates enzymatic browning and phenolic loss upon tissue disruption; and (iv) logistics of collecting and stabilizing a perishable, water-rich feedstock during a narrow sugar-campaign window [3,4]. These issues underscore the need for mild, selective process conditions and rapid stabilization to preserve phenolic integrity while meeting food/feed safety constraints. In this article, we focused on the first limiting factor, reducing this uncertainty by presenting compositional variability across cultivars, harvest dates, and leaf tissues, thereby defining practical guidance on when and what to collect to support targeted fractionation and stabilization of SBL streams.

Current literature points to several knowledge gaps specific to SBL. First, reported phenolic profiles and total polyphenol contents vary widely across studies, reflecting differences in cultivars, growth stage, agronomy, and analytical methods [4,6,10]. Second, systematic assessments of inter-seasonal and varietal variability in SBL phenolic compounds remain sparse, limiting robust specifications for process design. Third, while integrated routes that co-recover phenolic compounds and proteins are conceptually attractive, quantitative trade-offs between phenolic retention, antioxidant performance, and protein concentrate purity/functionality are not yet fully resolved at pilot-relevant scales [10,11,12,13,14].

From a sustainability perspective, valorizing SBL could diversify revenues at the farm-to-factory interface. Appropriate logistics and application solutions enabling the extraction and production of natural bioactive compounds based on SBL would result in beneficial changes in the form of effective and sustainable development of the sugar industry. Delivering such outcomes requires a combined view of composition, processing, and function, i.e., understanding how mild operating variables (pH, temperature, residence time, and oxygen exposure) govern phenolic recovery, stability, and bioactivity, while yielding protein fractions with target techno-functional attributes [4,14].

In the present manuscript, it is hypothesized that the compositional characteristics and related bioactive potential of sugar beet leaves are not constant but are primarily driven by leaf tissue fraction (leaf blades versus petioles), harvest time/phenological stage, and cultivar. Specifically, it is hypothesized that leaf blades accumulate higher levels of total polyphenols and major C-glycosyl flavones (vitexin derivatives) than petioles and that advancing plant development is associated with systematic shifts in polyphenol concentration.

Aim of the study: In this work, (i) total polyphenols are quantified and the phenolic composition of SBL is characterized using complementary HPLC-DAD and LC-MS methods; (ii) extraction routes enabling the recovery of a phenolic-rich extract and protein from the same biomass are developed and compared; and (iii) the distribution of polyphenol and protein content between leaf blades and petioles across different sugar beet cultivars and harvest times is evaluated.

## 2. Results and Discussion

### 2.1. Selection of the Optimal Extraction Solvent

The obtained results indicated that both the type of solvent and its concentration had a significant impact on the efficiency of polyphenol extraction in order to optimize the extraction stage.

The total polyphenol content of various extracts was analyzed using the HPLC-DAD method and expressed as vitexin equivalents (Table 1).

The highest total polyphenol content was obtained using 70% methanol as the solvent. In the case of the leaf blades, the value reached 11.7 mg/g DW, while for the entire leaf it was 12.3 mg/g DW. These results were higher than those achieved with ethanol at comparable concentrations, demonstrating greater effectiveness of methanol as an extraction solvent for polyphenols. In contrast, for the leaf petioles, the highest polyphenol content (0.6 mg/g DW) was recorded for extraction with 70 and 60% methanol and ethanol. This may result from differences in the chemical composition of individual leaf parts and the varying polarity of phenolic compounds present in the petioles. For both methanol and ethanol, an increase in solvent concentration up to 70% generally improved the extraction efficiency. However, in the case of ethanol, increasing the concentration above 60% did not lead to a further significant enhancement of extraction yield, unlike methanol.

Extraction using 70% MeOH as the extraction solvent was used to evaluate the polyphenol content in all analyzed SBL samples (blades and petioles).

### 2.2. Dry Weight Content

The analysis of dry weight (DW) content in sugar beet petioles and blades collected in August, September, and October revealed statistically significant differences between plant organs, cultivars, and sampling periods. According to the dataset presented in Table 2, the mean DW values for blades were 17.5% (range 14.5–20.6%) in August, 19.8% (16.3–23.9%) in September, and 20.2% (14.8–19.7%) in October.

Petioles exhibited systematically lower dry matter contents of 13.8%, 15.1%, and 13.9% for the same respective months. These results indicate that leaf blades generally accumulate more dry matter than petioles, and that the DW content increases from August to September, remaining stable thereafter. This trend is consistent with the physiological redistribution of assimilates during the later stages of sugar beet growth, when carbon allocation favors storage organs and older leaves undergo partial senescence accompanied by higher proportions of structural and phenolic compounds [3]. The obtained DW values for sugar beet blades are slightly higher than the lower range reported for fresh leaves in the literature, which typically spans 10–16% depending on genotype, growth stage, and environmental conditions. Vissers et al. [3] reported similar variability in sugar beet leaves, indicating that older leaves exhibit intensified phenolic metabolism, including increased synthesis and enzymatic oxidation of phenolic compounds, which may contribute to higher dry matter (DW) content. The values reported for fresh SBL biomass by Ebrahimi et al. [15] amounted to about 18%. The dry matter of the fresh sugar beet reported by Dukić et al. [16] was 22.97 ± 0.32%.

Simultaneously, genotypic variability among cultivars has been demonstrated to significantly influence dry matter (DW) deposition in sugar beet leaves. Genetic background affects assimilate partitioning and the balance between structural and non-structural carbohydrates, resulting in cultivar-specific differences in DW content under similar growth conditions [15,17]. Differences in dry matter accumulation under water and nutrient treatments have been documented in sugar beet [18,19]. Water availability influences dry matter partitioning under drought stress, reducing relative water content and altering biomass allocation [20,21], while nitrogen supply can significantly modulate DW outcomes by increasing soluble nitrogenous compounds and affecting carbohydrate deposition [18,19].

Taken together, these genetic and environmental factors interact to shape the variability in DW values observed among cultivars and across growing conditions. Enhanced understanding of these influences is critical for interpreting differences in biomass composition and for optimizing management practices in sugar beet cultivation.

### 2.3. Protein Content

**Figure 1:**
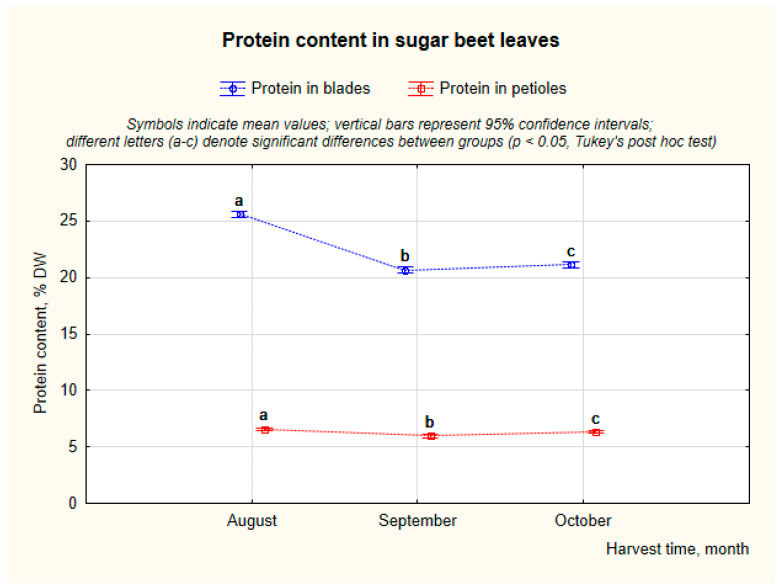
The content of protein in blades and petioles of SBL in % DW.

Leaf blades exhibited protein content (Figure 1) ranging from approximately 19% DW to 29% DW. In sugar beet leaf blades, protein content was significantly affected by harvest date. Post hoc comparisons indicated three distinct homogeneous groups, with the lowest mean value in September (20.7% DW), a slightly higher level in October (21.2% DW), and the highest content in August (25.6% DW). Individual cultivars such as ‘Smart Latoria’ exhibited exceptionally high statistical levels compared to others, reaching its maximum in October (29.6% DW), which may reflect genotype-specific accumulation or local environmental factors. Petioles showed a significantly lower protein content, typically ranging from 4.9% DW to 9.5% DW. Very small but statistically significant differences were observed between the different harvest months.

The obtained results showed a distinct differentiation of protein content between leaf fractions and across sampling periods. The higher protein content in leaf blades compared to petioles confirmed that mesophyll tissues are the primary reservoir of soluble proteins, while petioles contain mainly structural and carbohydrate-rich tissues with a low nitrogen content. The seasonal trend, a gradual decline from August to September, suggests a redistribution of nitrogen compounds from the leaves to the storage root during the later growth stages, consistent with the physiological transition toward carbohydrate accumulation.

The protein contents were comparable to the range of literature data reported for sugar beet leaves and other green plant tissues. Recent studies report protein levels in dried sugar beet leaves typically between 18% and 29% (DW basis), depending on genotype and agronomic factors [22,23,24,25]. Higher values, exceeding 30%, have occasionally been reported for concentrated protein fractions or enzymatically extracted preparations [15,26]. Seasonal decline in total protein also reflects well-known nitrogen remobilization patterns in sugar beet leaves, which have been observed in several studies. For example, Biondo et al. [27] reported that leaf protein content in *Beta vulgaris* decreased from early to later developmental stages (60 to 100 days), indicating a redistribution of nitrogenous compounds as leaves mature and nitrogen is remobilized to other plant parts [27]. Such trends are consistent with documented changes in nitrogen and protein pools during leaf ageing and senescence in sugar beet and related species, where both soluble protein and total nitrogen decline as leaves transition from active growth to nutrient export phases.

Sugar beet leaves constitute a protein-rich biomass, particularly in the blade fraction, and underline the potential of sugar beet leaf material as a valuable protein source for biorefinery or feed applications [14].

### 2.4. Polyphenols in Sugar Beet Leaves

#### 2.4.1. Characterization of Polyphenolic Compounds Identified in SBL by LC-MS

The LC/MS analyses of methanolic extracts of lyophilized sugar beet leaves revealed a profile of polyphenolic constituents primarily composed of flavonoid glycosides and phenolic acids. Twelve compounds were tentatively identified based on their retention times, UV–VIS spectra, molecular ions [*M*−*H*]^−^, and diagnostic fragment ions obtained in MS/MS mode.

Among the detected metabolites, flavones and flavonols were the predominant groups (Table 3). The UV chromatogram at the wavelength of 360 nm, showing the peaks from Table 3, and the MS/MS spectra of individual peaks from this table are provided in Supplementary Materials.

Several apigenin derivatives were identified, including apigenin dihexoside (*m*/*z* 593), apigenin hexopentoside (*m*/*z* 563), and two acylated apigenin hexopentosides (*m*/*z* 605). The fragmentation ions at *m*/*z* 413 and 293 and UV–VIS absorption maxima near 269–339 nm confirmed the presence of apigenin as the aglycone moiety. Additionally, characteristic loss of mass 120 is typical for apigenin dihexoside [28]. The acylated derivatives showed additional fragments consistent with ferulic or *p*-coumaric residues, suggesting the presence of acyl substituents typical for plant defense-related modifications. In addition, vitexin (apigenin-8-C-glucoside, *m*/*z* 431) was detected, indicating the coexistence of both O- and C-glycosylated apigenin forms in the leaf tissue.

The flavonol fraction was dominated by (iso)rhamnetin-based glycosides such as rhamnetin dihexoside (*m*/*z* 639) and rhamnetin hexopentoside (*m*/*z* 609), together with several unidentified flavonol dihexosides (*m*/*z* 635). In the case of all flavonol dihexosides (*m*/*z*), the loss of the hexose residue results in the formation of a characteristic fragment with *m*/*z* 473 in the MS/MS spectrum [28]. Fragment ions at *m*/*z* 315 and UV absorption maxima between 335 and 352 nm correspond to rhamnetin aglycone, a methylated derivative of quercetin commonly reported in *Beta* species and coming from the loss of sugar moieties. In the case of peaks 10 and 11 from Table 3, where *m*/*z* is 605, a subsequent loss of sugar residues is visible in the MS/MS spectrum (605 − 162 = 443—loss of hexose residue; 443 − 132 = 311—loss of pentose residue). The occurrence of multiple isomers differing in retention time implies structural diversity related to sugar composition and linkage position. Such structural heterogeneity of flavonol glycosides is often associated with differences in bioavailability and biological activity, including antioxidant and anti-inflammatory properties.

In addition to flavonoids, one phenolic acid derivative, diferulic acid (*m*/*z* 387), was also identified. Its fragmentation ions at *m*/*z* 289 and 193, together with a UV maximum near 326 nm, are characteristic. Diferulic acid participates in the formation of covalent linkages in plant cell walls, contributing to mechanical resistance and oxidative stress protection.

The results demonstrate that sugar beet leaves are a rich source of polyphenolic compounds, particularly glycosylated flavones and flavonols, with apigenin and isorhamnetin as dominant aglycones and the presence of C-glycosylated derivatives such as vitexin. The presence of acylated and mixed glycosides suggests cross-linked ferulate dimers complex secondary metabolism pathways related to photoprotection and stress adaptation.

According to recent studies [4,23] on sugar beet leaves, the flavone C-glycoside vitexin is the quantitatively dominant individual phenolic compound, although phenolic acids (e.g., ferulic and gallic acids) and other flavonoids also substantially contribute to the overall polyphenol pool.

These findings align with previous reports on *Beta vulgaris* phenolic composition and indicate a high polyphenolic content that may be associated with antioxidant activity, suggesting that the leaf fraction could be a valuable by-product for nutraceutical and functional food applications.

#### 2.4.2. Total Polyphenol Content in SBL

Since vitexin was identified as the predominant individual phenolic compound in sugar beet leaves, the total polyphenol content (TPC), the sum of all phenolic peaks, was quantified using vitexin as an external standard. The average TPC (Table 4) in SBL blades was 9.7 mg/g DW (SD 2.67). The highest TPC was found in the ‘Wojownik’, ‘Jagienka’, ‘Mariza’, and ‘Smart Latoria’ varieties (approx. 10–11 mg/g DW). The lowest values were found in the ‘Jantar’ and ‘Jagiellon’ varieties (below 9 mg/g DW).

Statistically significant differences were found between the varieties, and the ‘Wojownik’ and ‘Jagienka’ varieties may have higher leaf antioxidant potential. SBL petioles had a significantly lower mean polyphenol content compared to blades, amounting to 1.5 mg/g DW, with a standard deviation of 0.73. The highest concentrations were found in the ‘Smart Latoria’ and ‘Mariza’ varieties (2.7 mg/g DW and 2.2 mg/g DW, respectively), and the lowest in the ‘Jagienka’, ‘Wojownik’, and ‘Jagiellon’ varieties (approx. 0.8 mg/g DW).

The highest polyphenol content in blades was recorded during the August harvest, decreasing with harvest date (Table 5). This suggests that early harvests promote greater polyphenol accumulation, and their content decreased as the plants matured. Unlike blades, petioles show less variability and a slightly different trend; the maximum content was recorded in September, which may be due to a shift in phenolic metabolism between plant parts. Polyphenol content in blades strongly depends on harvest date, while in petioles this factor is less affected, but there are clear differences between cultivars.

**Figure 2:**
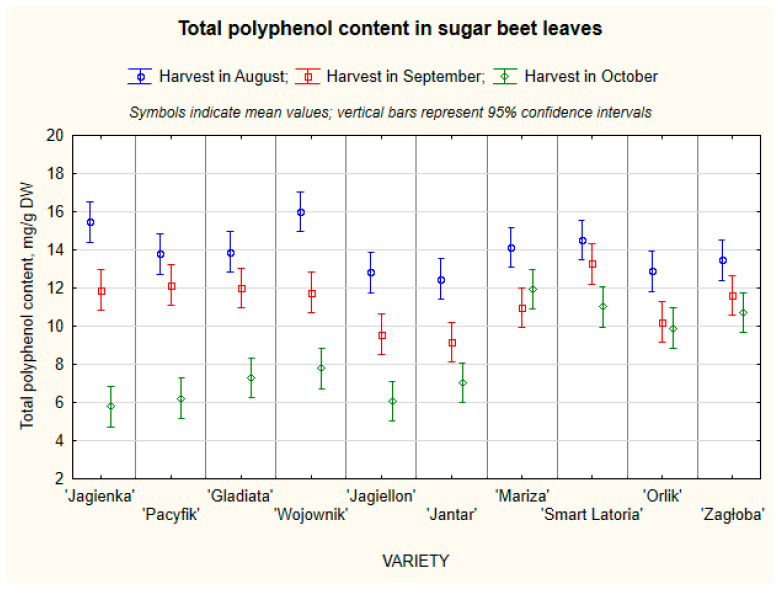
The content of total polyphenol in SBL.

Significant genotypic variability was observed among the tested varieties, with polyphenol contents in whole leaves ranging from 9.48 mg/g DW (‘Jagiellon’) to 12.93 mg/g DW (’Smart Latoria’) (Figure 2).

The highest concentrations were found in the varieties ‘Smart Latoria’, ‘Mariza’, and ‘Zagłoba’, whereas the lowest values occurred in ‘Jagiellon’ and ‘Jantar’. Such variation may reflect differences in metabolic activity and secondary metabolite pathways among genotypes of plants.

When compared with literature data, the total phenolic content determined in this study aligns with or slightly exceeds the values reported for sugar beet leaves extracted under similar conditions. Ebrahimi et al. [4] reported an average total phenolic content of 6.8–17.2 mg/g DW for various extraction techniques and up to 69.4 mg/g DW using ultrasound assisted extraction, depending on solvent composition and extraction parameters. Similarly, Maravić et al. [29] found 4.5–17.2 mg/g DW in dried leaves using ethanol extraction and Dukić et al. [16] found values from 13 to 18 mg/g DW, whereas El-Gengaihi et al. [29] observed lower concentrations of 1.6–16.1 mg/g DW.

The predominant phenolic compound class in sugar beet leaves, as highlighted by Ebrahimi et al. [4] and Maravić et al. [29], includes flavonoids such as vitexin, isovitexin, and catechin derivatives, alongside phenolic acids like ferulic and *p*-coumaric acid. These compounds contribute to strong antioxidant properties and have been linked to hepatoprotective and anti-inflammatory activities. Variations in polyphenol content across studies may arise from genotype-dependent expression of biosynthetic enzymes, environmental stress conditions, and growth stage at harvest [27].

Overall, the results indicate that the sugar beet leaves analyzed in this study represent a valuable source of polyphenolic antioxidants, with levels comparable to those reported in the recent literature. These findings support the growing evidence that sugar beet leaves, traditionally considered an agricultural by-product, can be valorized as a promising raw material for obtaining high-value phenolic compounds, aligning with current biorefinery and sustainability concepts.

A pronounced seasonal effect was also evident. Mean polyphenol levels declined progressively from August (13.94 mg/g DW) to October (8.37 mg/g DW). This trend suggests that early-harvest leaves are richer in phenolic compounds, likely due to more active biosynthesis and lower oxidative degradation in younger tissues.

Seasonal changes in foliar phenolics have been reported for sugar beet leaves, with plant age affecting total phenolic content and browning behavior [3]. The interaction between variety and harvest time further supports this pattern: early harvest samples of varieties such as ‘Wojownik’ and ‘Jagienka’ exhibited the highest phenolic concentrations (up to 15.9 mg/g DW), while late harvest samples from the same varieties showed substantial decreases.

When compared with published data, the present values (8–15 mg/g DW, vitexin equivalents) fall within or slightly above the upper range reported for *Beta vulgaris* leaves. The differences in polyphenol values obtained here likely reflect the use of different extraction methodologies, different methods of analysis, and different reference compounds (vitexin instead of gallic acid), which typically yields more conservative but chemically specific quantification [4]. Moreover, discrepancies may also arise from whether entire leaves are included in the analysis or only the leaf blades, with petioles omitted, as these plant parts differ in their polyphenolic composition. Such methodological differences highlight the importance of comparing results within the same analytical framework.

#### 2.4.3. Vitexin and Vitexin Derivatives Content in SBL

Vitexin (apigenin-8-C-glucoside) is a widely distributed flavone C-glycoside occurring in numerous medicinal and food plants [30]. High levels of vitexin and its derivatives have been reported in passionflower (*Passiflora incarnata*), hawthorn (*Crataegus* spp.), chaste tree (*Vitex agnus-castus*), and bamboo leaves, as well as in several cereals and pseudocereals such as millet and buckwheat [31]. Recent studies also indicate that sugar beet (*Beta vulgaris*), particularly its leaves, may represent an additional, underutilized source of vitexin for further utilization for example in the human diet [32]. Due to this multifunctional bioactivity, vitexin is increasingly regarded as a promising compound for nutraceutical and functional food applications, as well as a lead structure in the development of phytopharmaceuticals aimed at supporting cardiovascular health, neuroprotection, and metabolic homeostasis [33,34,35].

**Figure 3:**
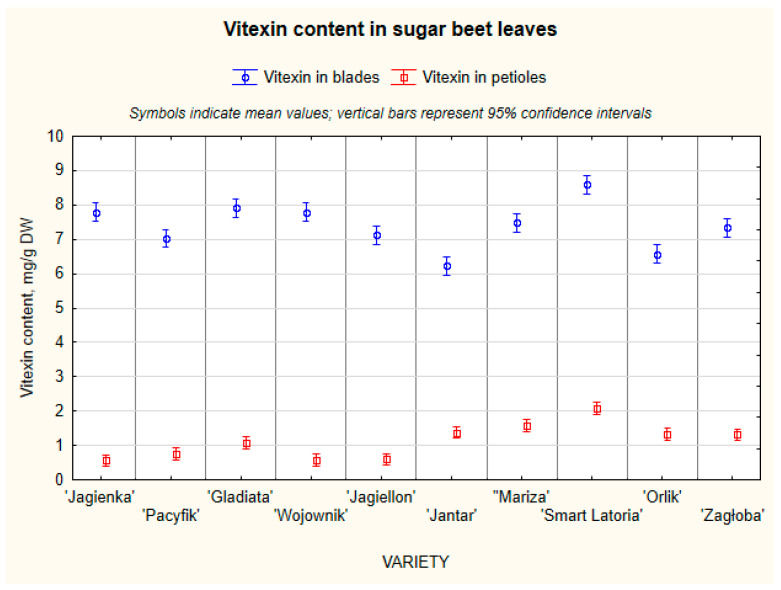
The content of vitexin in different various SBL.

As presented in this study, leaf blades contained substantially higher concentrations of vitexin (mean 7.4 ± 2.3 mg/g DW) than petioles (1.1 ± 0.6 mg/g DW) (Figure 3). This is consistent with the structural and physiological specialization of leaf tissues: leaf blades accumulate higher concentrations of photoprotective flavonoids. Comparable tissue-dependent phenolic distributions in sugar beet have been documented by Vissers et al. [3]. The ‘Smart Latoria’ variety exhibited the highest mean vitexin concentration (10.6 mg/g DW), while ‘Jantar’ showed the lowest (6.2 mg/d DW). This study indicates that genetic factors influence vitexin accumulation, but the effects may be moderated by environmental or physiological variability.

**Figure 4:**
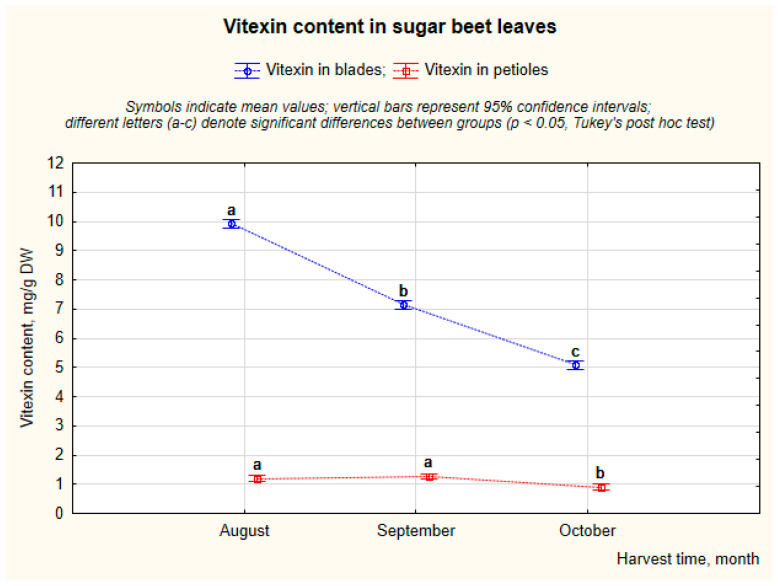
Variation in vitexin content depending on harvest time.

Harvest timing markedly affected vitexin accumulation; the highest concentrations measured in August (9.9 mg/g DW), and the lowest in October (5.1 mg/g DW) (Figure 4). This trend reflects leaf maturation and reduced flavonoid biosynthesis later in the season, as also reported in other studies examining seasonal or developmental changes in phenolic compounds in plants [36,37]. The significant interaction between harvest time and part of leaf demonstrates that leaf blades exhibited a sharper seasonal decrease than petioles.

The percentage contribution of vitexin derivatives to total polyphenol content (TPC) was high across all samples (above 70%) and decreased from August (80.1%) to October (72.5%). No significant variation in the percentage contribution of vitexin derivatives to TPC was observed between blades and petioles; although the total phenolic content is much higher in blades, the relative composition of phenolic compounds remains stable between tissues.

The results demonstrate that leaf blades and early-season harvests of sugar beet leaves are optimal for maximizing vitexin content, which is consistent with biochemical studies describing the regulation of C-glycosyl flavonoid biosynthesis in leaves and emphasizes the potential for optimizing harvest and processing strategies for vitexin-rich plant material.

## 3. Materials and Methods

### 3.1. Plant Material

**Figure 5:**
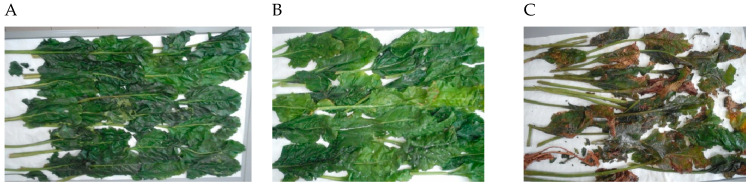
Photos of sugar beet leaves ‘Jagiellon’ varieties harvested at the 1st of August (**A**), the 3rd of September (**B**), and the 13th of October (**C**).

The research material included sugar beet leaves from ten sugar beet varieties: collected in 2021 on three different harvest dates: 1st of August, 3rd of September, and 13th of October and collected in 2022 on the 1st of August, 7th of September, and 1st of October. Sugar beet leaf varieties collected: In 2021, ‘Jagienka’ (Kutno Sugar Beet Breading Farm, KHBC, Kutno, Poland), ‘Pacyfik’ (Maribo^®^, Holeby, Denmark), ‘Gladiata’ (KWS, Einbeck, Germany), ‘Wojownik’ (SESVanderHave, Calignac, France), and ‘Jagiellon’ (Wielkopolska Sugar Beet Farm WHBC, Poznań, Poland). In 2022, ‘Jantar’ (KHBC, Straszków, Poland), ‘Mariza’ (Maribo^®^, Holeby, Denmark), ‘Smart Latoria’ (KWS, Einbeck, Germany), ‘Orlik’ (SESVanderHave, Calignac, France), and ‘Zagłoba’ (WHBC, Poznań, Poland). The leaves were harvested from the experimental field in Poland. The research material, in the laboratory, was divided into sugar beet leaf blades and petioles. To preserve the material, the sugar beet leaves blades and sugar beet leaves petioles (separately) was frozen in liquid nitrogen and ground using an IKA Basic cryogenic mill (IKA-Werke GmbH & Co. KG, Staufen, Germany), then freeze-dried under reduced pressure (0.1 bar) at −50 °C (Martin Christ Gefriertrocknungsanlagen GmbH, Osterode am Harz, Germany). The resulting powder constituted the homogeneous research material and was stored in a polypropylene container (DENIOS SE, Bad Oeynhausen, Germany) with a desiccant (Merck KGaA, Darmstadt, Germany). Figure 5 shows representative photos of leaves from the ‘Jagienka’ variety, collected at different harvest dates (August, September, and October).

### 3.2. Extraction of Polyphenols

Before the entire research material was analyzed, one selected variety of beet leaves was subjected to preliminary extraction in order to select the extraction solvent and its concentration. The ‘Jagiellon’ variety was extracted using methanol (MeOH; ≥99.9%, gradient grade, for HPLC, Sigma-Aldrich^®^, Steinheim, Germany) and ethanol (EtOH; ≥99.9%, gradient grade, for HPLC, Sigma-Aldrich^®^, Steinheim, Germany) at various concentrations (50%, 60%, and 70%, *v*/*v*) containing 0.1% formic acid (*v*/*v*) (98–100%, for LC-MS LiChropu, Merck KGaA, Darmstadt, Germany).

The extraction process took place in an ultrasonic bath (Sonic-10, Polsonic, Warsaw, Poland) at room temperature. In total, 0.5 g of leaf blades or leaf petioles was mixed with 3 mL of extraction solvent for 15 min. The extraction process was repeated three times. After each extraction, the mixture was centrifuged (MPW-260H, MPW Med. Instruments, Warsaw, Poland) for 10 min at 12,000× *g* and the supernatant was decanted. The collected supernatant was transferred quantitatively to a 10 mL graduated flask and topped up with extraction solvent.

### 3.3. Chromatographic Analysis

The extracts were analyzed using high-performance liquid chromatography with a diode-array spectrophotometric detector (HPLC–DAD, Thermo Scientific Dionex, Waltham, MA, USA) and using high-performance liquid chromatography with a mass spectrophotometer (HPLC-MS, LCQ DECA, Thermo Fisher Scientific, Waltham, MA, USA), equipped with an ESI source in the negative mode, to identify polyphenolic compounds. Separation and identification of polyphenolics was conducted with the Phenomenex Luna 5 μm C18 column (250 × 4.6 mm). The extracts were filtered through membrane filters with a pore diameter of 0.45 mm before injection.

The separation was conducted in the following conditions: flow—0.5 mL min^−1^; column temperature—30 °C; injection volume—20 µL. Mobile phase A consisted of 1% formic acid (98–100%, for LC-MS LiChropu, Merck KGaA, Darmstadt, Germany) in water, and mobile phase B was 0.5% formic acid (98–100%, for LC-MS LiChropu, Merck KGaA, Darmstadt, Germany) in 80% acetonitrile (≥99.8%, HPLC grade, Supelco^®^, Fresno, CA, USA) (80:19.5:0.5, ACN:H_2_O:HCOOH, *v*/*v*/*v*). The separation gradient was as follows: 0–6 min, 4% (*v*/*v*) B; 6.5–12.5 min, 4–12% (*v*/*v*) B; 12.5–44 min, 12–36% (*v*/*v*) B; 44–45 min, 36–60% (*v*/*v*) B; 45–50 min, 60% (*v*/*v*) B, 50–52 min, 60–4% (*v*/*v*) B; and 52–65 min, 4% (*v*/*v*) B.

The DAD detector (Thermo Fisher Scientific, Waltham, MA, USA) recorded spectra simultaneously in the range of 200–600 nm, and the mass spectrometer recorded spectra in negative mode. The ion source parameters were set as follows: vaporizer temperature, 500 °C; ion spray voltage, 4 kV; capillary temperature, 400 °C; sheath gas flow rates, 75 arbitrary units. The MS/MS data was generated using helium gas to fragment precursor ions. In full MS mode, the scan range of *m*/*z* 150–2000 was used. To generate MS2 data, the full MS/dd-MS2 scan mode was used. In this mode, the selected precursor ions entered into an HDC collision cell, where they were fragmented with normalized collision energy (NCE) to obtain product ion spectra (MS2). In these experiments, the NCE used to generate MS2 spectra was set to 20. Data were collected using the Chromeleon^®^ software version 6.70 (Dionex, Bellefonte, PA, USA) and the Xcalibur software version 1.2 (Thermo Fisher Scientific, Waltham, MA, USA).

Polyphenolic compounds were identified by LC-MS/MS (Thermo Fisher Scientific, Waltham, MA, USA), and each detected peak was quantified individually using vitexin as an external standard. The concentrations of all identified polyphenols were subsequently summed and reported as the total polyphenol content of the analyzed material. The calibration curve of vitexin standard and validation parameters are presented in Supplementary Materials (Figure S15 and Table S1).

### 3.4. Dry Weight

Dry weight (DW) content was assessed by drying the samples at 105 °C until a constant mass was achieved. This widely used gravimetric method relies on the removal of water through evaporation from a precisely weighed portion of the sample. The dry matter value is obtained from the change in sample mass before and after the drying process.

### 3.5. Protein

The total protein content was determined using the Kjeldahl method (AOAC 2001.11), which measures the nitrogen in the samples and estimates the total protein. A nitrogen-to-protein conversion factor of 6.25 was applied. The calculated protein content is expressed as a percentage; the percentage values refer to the total weight of the sample of the sample [38].

### 3.6. Statistical Analysis

All chemical analyses were performed in triplicate. The data are presented as the means ± SDs from three analytical replicates of independent experiments (*n* = 3). Data were subjected to basic statistics of two-factor ANOVA analysis and post hoc Tukey’s (HSD) test for identification of differences between the groups. Statistical analyses were carried out using the STATISTICA data analysis software system, with a level of confidence of *p* < 0.05 [39].

## 4. Conclusions

The present study demonstrates the compositional variability and valorization potential of sugar beet leaf (SBL), a ubiquitous by-product of sugar beet harvesting that is largely left in the field despite its rich content of bioactive compounds. The primary motivation behind this research was to address the underutilization of SBL biomass by characterizing the distribution of total polyphenols and proteins in both leaf blades and petioles across multiple cultivars and harvest times and to highlight opportunities for their use in value-added applications (e.g., nutraceuticals and functional ingredients).

Mapping the distribution of polyphenol and protein contents between leaf blades and petioles across cultivars and harvest times, we showed that tissue type and phenological stage strongly shape the composition of SBL. Leaf blades consistently exhibited a higher concentration of total polyphenols and protein than petioles. Vitexin derivatives contributed above 70% of the total polyphenols in both leaf parts, with their proportion decreasing with delayed harvest. Cultivar had only a minor effect on the composition and the levels of the analyzed metabolites compared with the stronger influences of harvest time and leaf tissue fraction.

The demonstrated variability in protein and polyphenol profiles has important implications for the valorization of sugar beet foliage. High levels of phenolic compounds and proteins in sugar beet leaves suggest that this biomass could be exploited as a source of functional ingredients for food, feed, or biorefinery streams.

Based on these results, future work should focus on (i) identifying environmentally friendly, food-grade extraction solvents suitable for use in products intended for contact with food, which would allow for the recovery of the highest possible amounts of polyphenolic compounds from sugar beet leaves; (ii) assessing the biological activity of SBL extracts to validate health-related functionalities; and (iii) establishing pilot-scale valorization models that integrate crop residues into circular economy frameworks. Such efforts will help to bridge the gap between analytical characterization and practical application, ultimately supporting more sustainable use of agricultural biomass.

## Figures and Tables

**Table 1:** The average content (±SD) of total polyphenol content in petioles and blades of the ‘Jagiellon’ variety extracted using methanol (MeOH) and ethanol (EtOH) at various concentrations (50%, 60%, and 70%).

Total Polyphenol Content, mg/g DW						
	MeOH 70%	MeOH 60%	MeOH 50%	EtOH 70%	EtOH 60%	EtOH 50%
**blades**	11.7 ^a^ ± 0.4	11.0 ^a,b^ ± 0.1	10.4 ^b^ ± 0.5	8.3 ^c^ ± 0.2	8.9 ^b,c^ ± 0.9	6.3 ^d^ ± 0.5
**petioles**	0.6 ^a^ ± 0.07	0.6 ^a^ ± 0.03	0.4 ^b^ ± 0.03	0.6 ^a^ ± 0.11	0.6 ^a^ ± 0.18	0.4 ^b^ ± 0.06

^a,b,c,d^—significant differences between groups (*p* < 0.05, post hoc Tukey’s test).

**Table 2:** The average content of dry weight (DW) (±SD) in parts of sugar beet leaves (blades and petioles) collected in August, September, and October harvest.

	Dry Weight, %					
Harvest	August		September		October	
Varieties	Blades	Petioles	Blades	Petioles	Blades	Petioles
‘Jagienka’	15.3 ± 0.3	13.1 ± 0.2	16.3 ± 0.3	13.3 ± 0.2	18.9 ± 0.2	11.7 ± 0.3
‘Pacyfik’	15.6 ± 0.3	12.9 ± 0.3	18.5 ± 0.3	14.9 ± 0.2	19.7 ± 0.2	11.8 ± 0.2
‘Gladiata’	14.5 ± 0.2	12.4 ± 0.3	17.5 ± 0.3	18.2 ± 0.3	17.4 ± 0.2	13.1 ± 0.2
‘Wojownik’	15.1 ± 0.3	12.9 ± 0.2	17.6 ± 0.2	13.1 ± 0.2	16.2 ± 0.2	11.8 ± 0.2
‘Jagiellon’	15.9 ± 0.7	12.3 ± 0.2	16.7 ± 0.8	12.6 ± 0.2	16.9 ± 0.6	10.6 ± 0.3
‘Jantar’	18.8 ± 0.2	21.7 ± 0.1	22.9 ± 0.2	14.3 ± 0.3	14.8 ± 0.2	15.8 ± 0.2
‘Mariza’	20.6 ± 0.2	23.9 ± 0.2	23.9 ± 0.2	15.3 ± 0.3	15.2 ±0.2	16.9 ± 0.2
‘Smart Latoria’	20.1 ± 0.2	21.7 ± 0.3	21.0 ± 0.2	14.5 ± 0.2	15.1 ± 0.3	15.3 ± 0.2
‘Orlik’	19.4 ± 0.3	22.2 ± 0.2	22.9 ± 0.3	15.1 ± 0.2	16.8 ± 0.3	16.1 ± 0.3
‘Zagłoba’	19.2 ± 0.4	22.2 ± 0.3	22.6 ± 0.3	14.8 ± 0.3	16.2 ± 0.2	16.1 ± 0.2

**Table 3:** The list of metabolites identified in sugar beet leaves extract via LC/MS technique.

No	t_R_ (min)	Tentative Identification	Nominal Mass (Da)	MS Data (*m*/*z*)	MS/MS Data (*m*/*z*)	UV-VIS max (nm)
1	27.1	apigenin dihexoside	594	[593]^−^	473, 413, 293	269, 339
2	28.1	apigenin hexopentoside	564	[563]^−^	413, 293	269, 328
3	28.7	(iso)rhamnetin hexopentoside	610	[609]^−^	285	270, 336
4	28.8	vitexin	432	[431]^−^	341, 311, 283	269, 338
5	29.0	flavonol dihexoside	636	[635]^−^	473, 413, 311, 293	268, 336
6	29.3	(iso)rhamnetin dihexoside	640	[639]^−^	315	270, 352
7	29.7	flavonol dihexoside	636	[635]^−^	593, 575, 515, 473, 413, 311, 293	268, 335
8	30.4	(iso)rhamnetin hexopentoside	610	[609]^−^	315	254, 363
9	30.5	flavonol dihexoside	636	[635]^−^	575, 473, 455, 329, 311, 293	270, 335
10	30.9	acylated apigenin hexopentoside	606	[605]^−^	563, 545, 455, 433, 413, 395, 353, 311, 293	269, 333
11	31.8	acylated apigenin hexopentoside	606	[605]^−^	545, 455, 443, 311, 293	270, 335
12	34.1	diferulic acid	388	[387]^−^ [193]^−2^	309, 289, 96 289, 193, 96	270, 326

**Table 4:** The average total polyphenol content (±SD) in SBL blades and petioles of various varieties.

VARIETY	TPC, mg/g DW Blades	TPC, mg/g DW Petioles
‘Jagienka’	10.3 ^a,b^ ± 4.2	0.8 ^a^ ± 0.3
‘Pacyfik’	9.7 ^a,c^ ± 3.2	1.0 ^a,c^ ± 0.4
‘Gladiata’	9.7 ^a,c^ ± 2.8	1.4 ^c,d^ ± 0.5
‘Wojownik’	11.0 ^b^ ± 3.6	0.8 ^a^ ± 0.3
‘Jagiellon’	8.7 ^c,d^ ± 2.9	0.8 ^a^ ± 0.3
‘Jantar’	7.8 ^d^ ± 2.2	1.7 ^b^ ± 0.3
‘Mariza’	10.2 ^a,b^ ± 1.4	2.2 ^e^ ± 0.3
‘Smart Latoria’	10.2 ^a,b^ ± 1.9	2.7 ^f^ ± 0.4
‘Orlik’	9.1 ^a,c^ ± 1.5	1.8 ^b,e^ ± 0.3
‘Zagłoba’	10.2 ^a,b^ ± 1.0	1.7 ^b,d^ ± 0.5

^a,b,c,d,e,f^—significant differences between groups (*p* < 0.05, post hoc Tukey’s test).

**Table 5:** The average total polyphenol content (±SD) in SBL blades and petioles at different harvest times.

HARVEST	Average Total Polyphenol Content, mg/g DW Blades	Average Total Polyphenol Content, mg/g DW Petioles
August	12.4 ^a^ ± 1.7	1.6 ^a^ ± 0.7
September	9.6 ^b^ ± 1.2	1.7 ^a^ ± 0.5
October	7.2 ^c^ ± 1.8	1.2 ^b^ ± 0.8

^a,b,c^—significant differences between groups (*p* < 0.05, post hoc Tukey’s test).

## Data Availability

The raw data supporting the conclusions of this article will be made available by the authors on request.

## Supplementary Materials

The following supporting information can be downloaded at: https://www.mdpi.com/article/10.3390/molecules31030489/s1, Figure S1: Example LC/MS chromatogram of SBL sample, Figure S2: The LC/MS chromatogram of Peak No 1, Figure S3: The LC/MS chromatogram of Peak No 2, Figure S4: The LC/MS chromatogram of Peak No 3, Figure S5: The LC/MS chromatogram of Peak No 4, Figure S6: The LC/MS chromatogram of Peak No 5, Figure S7: The LC/MS chromatogram of Peak No 6, Figure S8: The LC/MS chromatogram of Peak No 7, Figure S9: The LC/MS chromatogram of Peak No 8, Figure S10: The LC/MS chromatogram of Peak No 9, Figure S11: The LC/MS chromatogram of Peak No 10, Figure S12: The LC/MS chromatogram of Peak No 11, Figure S13: The LC/MS chromatogram of Peak No 12, Figure S14. Example chromatogram HPLC-DAD of SBL sample, Figure S15. Calibration curve, linearity and coefficient of determination (R^2^) of vitexin standard; Table S1: Validation parameters for the determination of vitexin standard.

## Abbreviations

The following abbreviations are used in this manuscript:

SBL	Sugar beet leaves
DW	Dry weight
TPC	Total polyphenol content
MeOH	Methanol
EtOH	Ethanol
