# Supplementary material for: Polyphenols in Sugar Beet Leaves: Composition, Variability, and Valorization Opportunities
Source: Molecules. 2026 Jan 30;31(3):489. doi: 10.3390/molecules31030489 (PMC12898648; doi:10.3390/molecules31030489)
Supplement: Supplementary file 1 [file molecules-31-00489-s001.zip › molecules-4044742-supplementary.pdf]

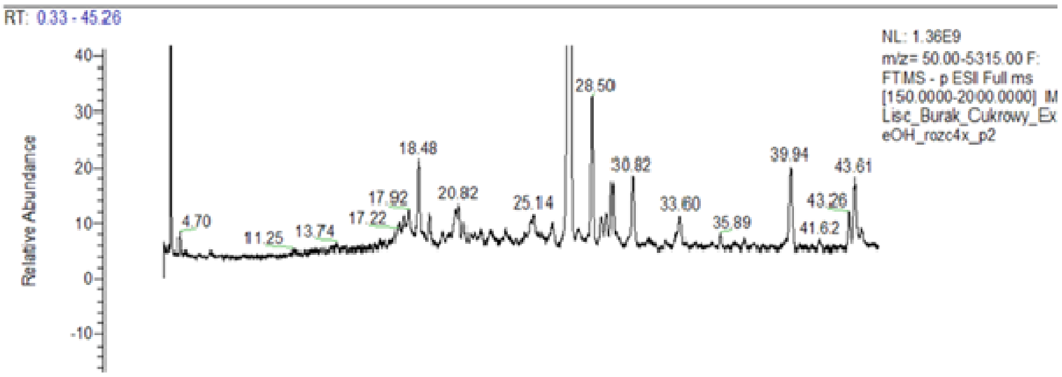

Figure S1. Example LC/MS chromatogram of SBL sample

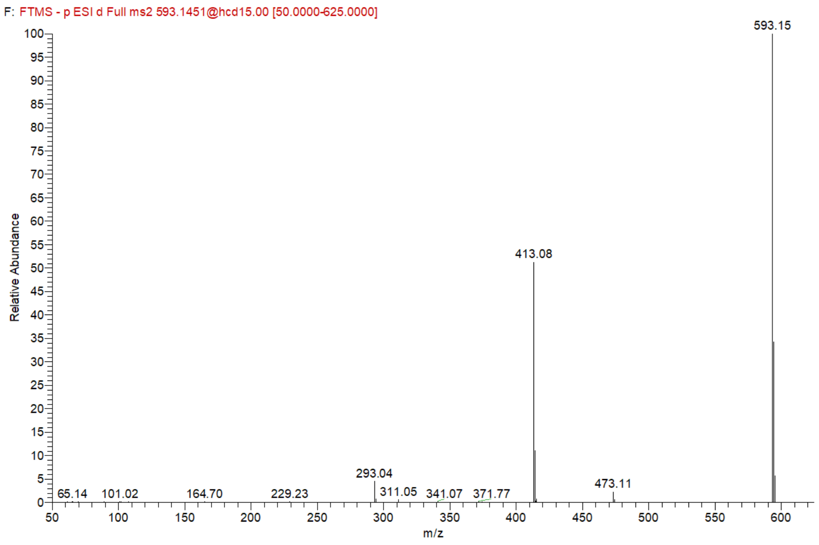

Figure S2. The LC/MS chromatogram of Peak No 1

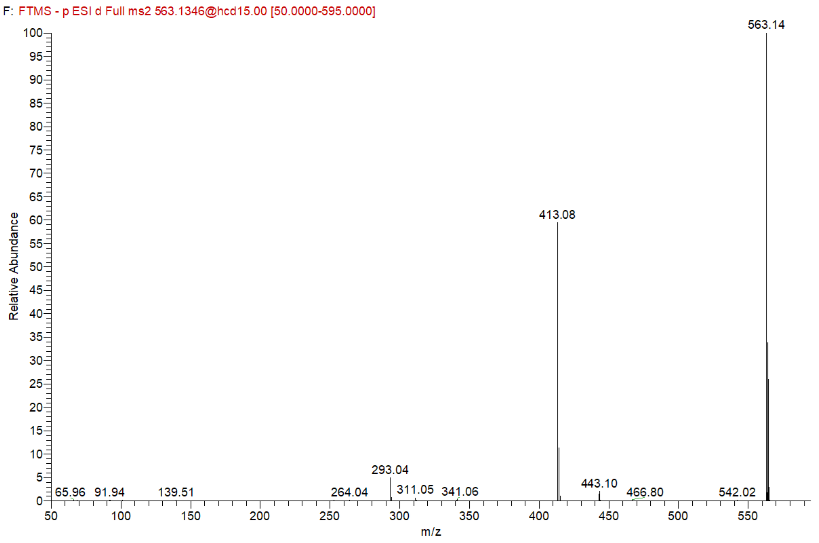

Figure S3. The LC/MS chromatogram of Peak No 2

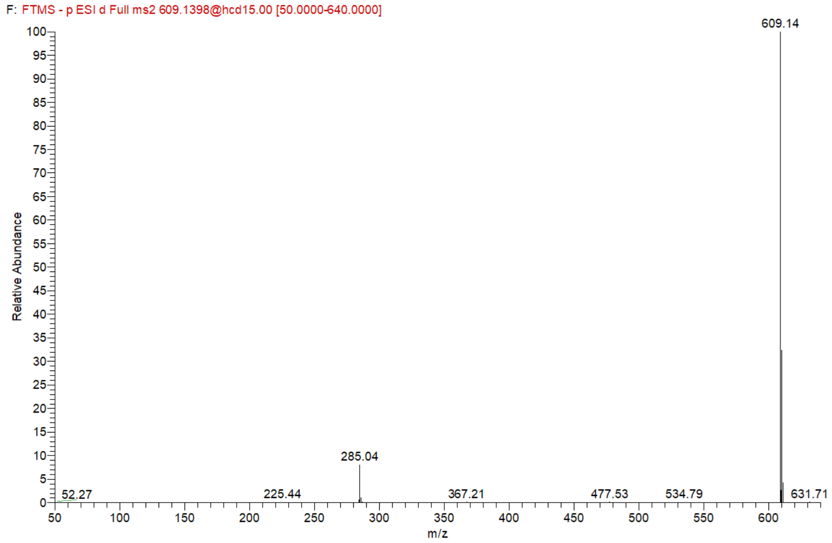

Figure S4. The LC/MS chromatogram of Peak No 3

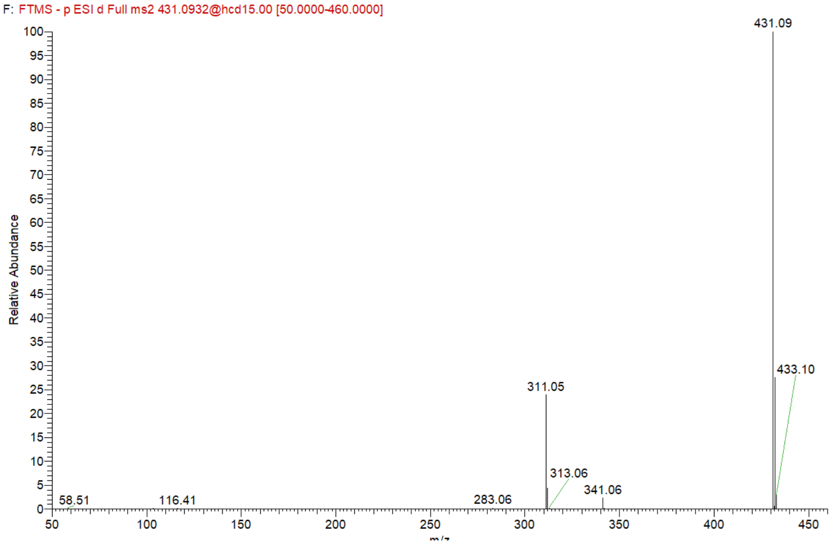

Figure S5. The LC/MS chromatogram of Peak No 4

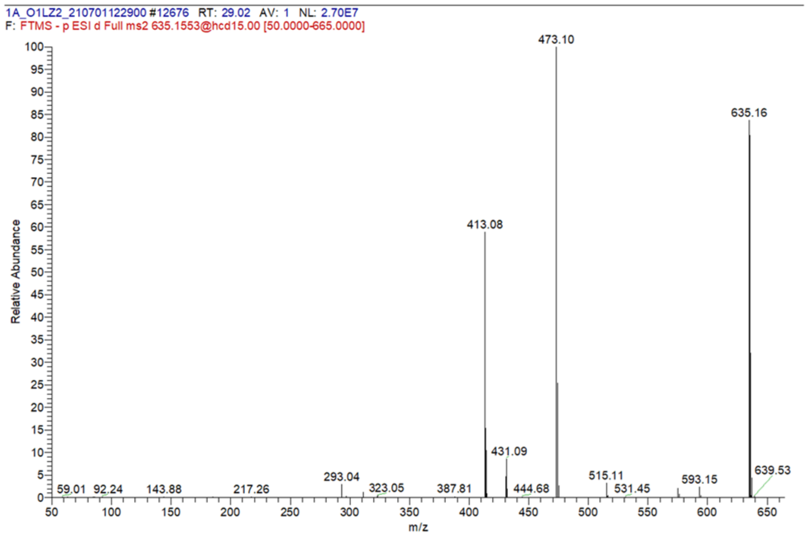

Figure S6. The LC/MS chromatogram of Peak No 5

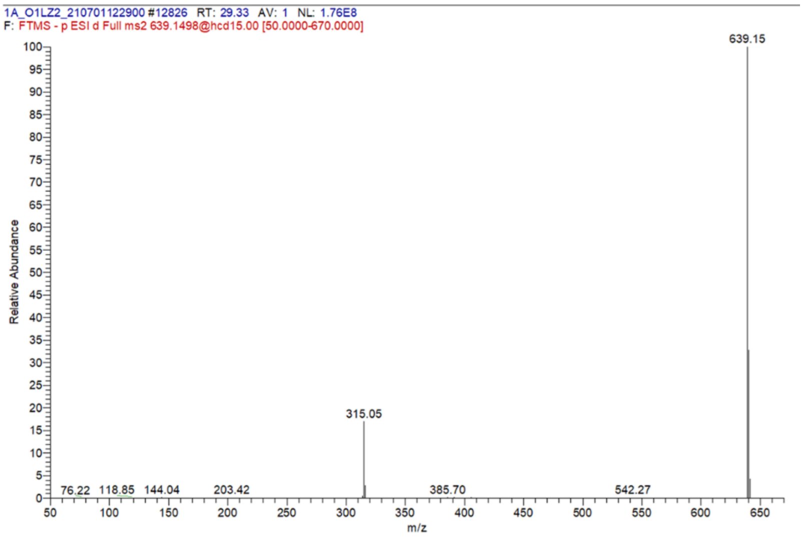

Figure S7. The LC/MS chromatogram of Peak No 6

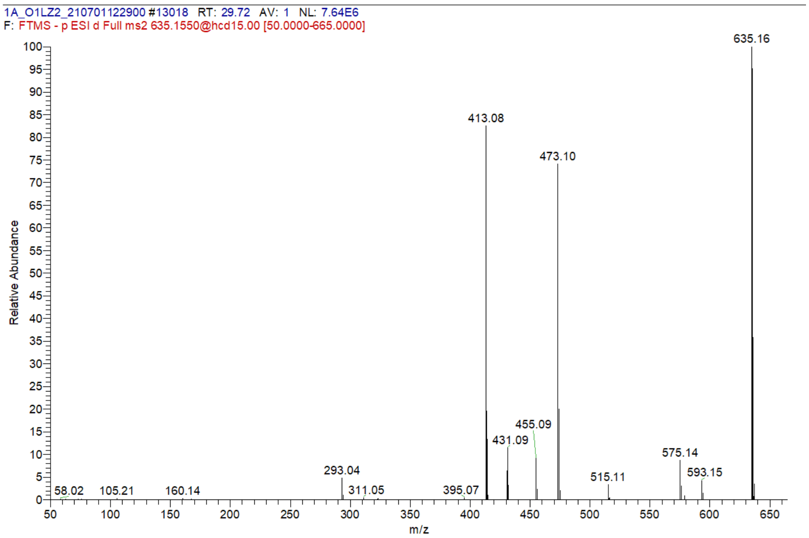

Figure S8. The LC/MS chromatogram of Peak No 7.

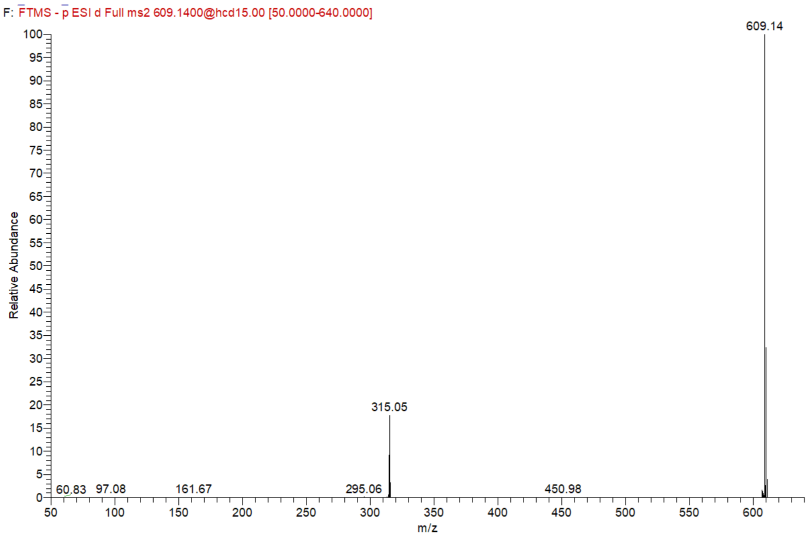

Figure S9. The LC/MS chromatogram of Peak No 8

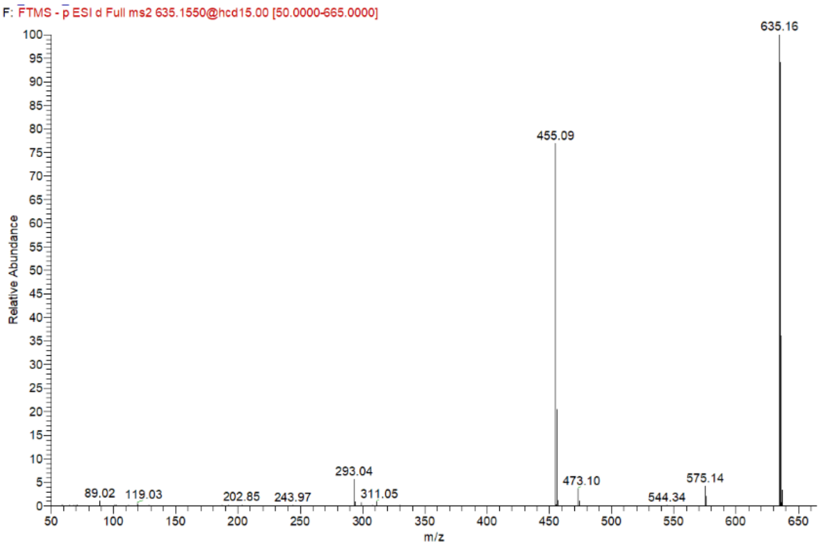

Figure S10. The LC/MS chromatogram of Peak No 9

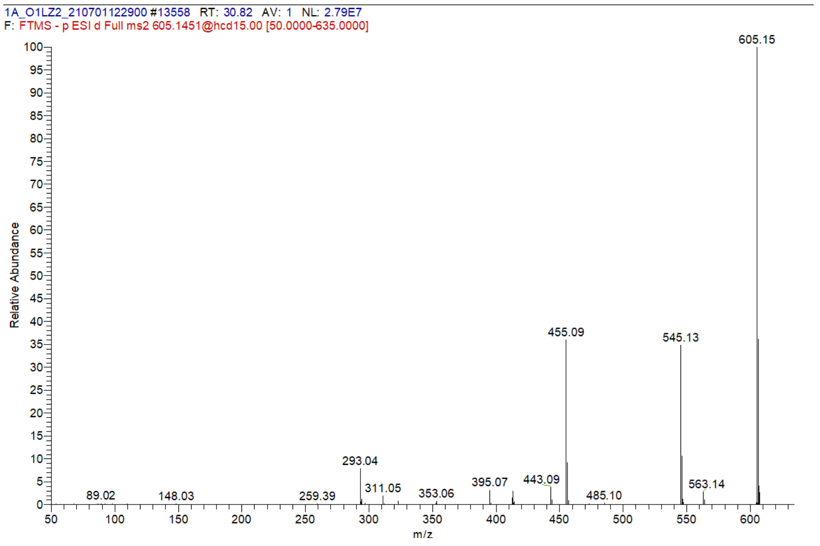

Figure S11. The LC/MS chromatogram of Peak No 10

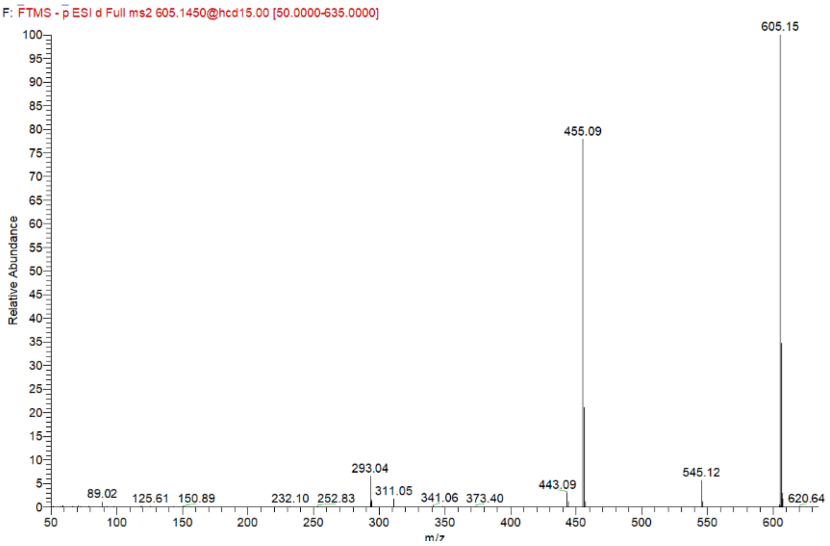

Figure S12. The LC/MS chromatogram of Peak No 11

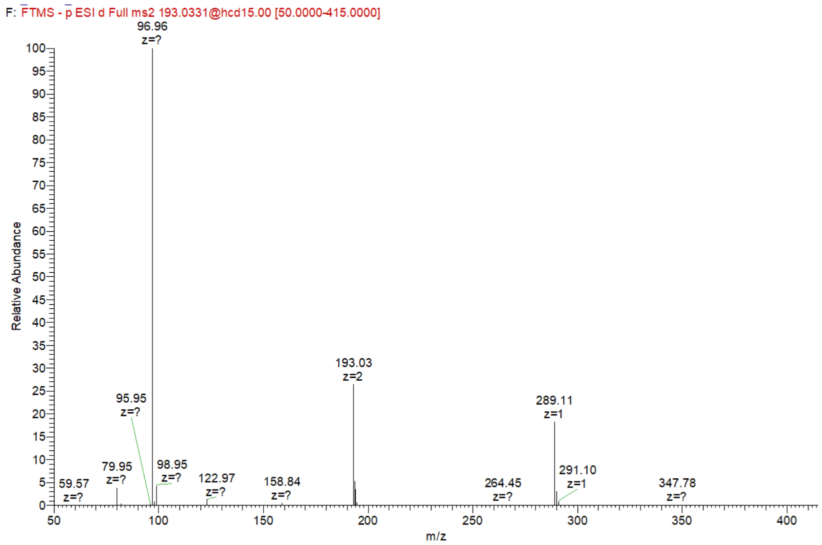

Figure S13. The LC/MS chromatogram of Peak No 12

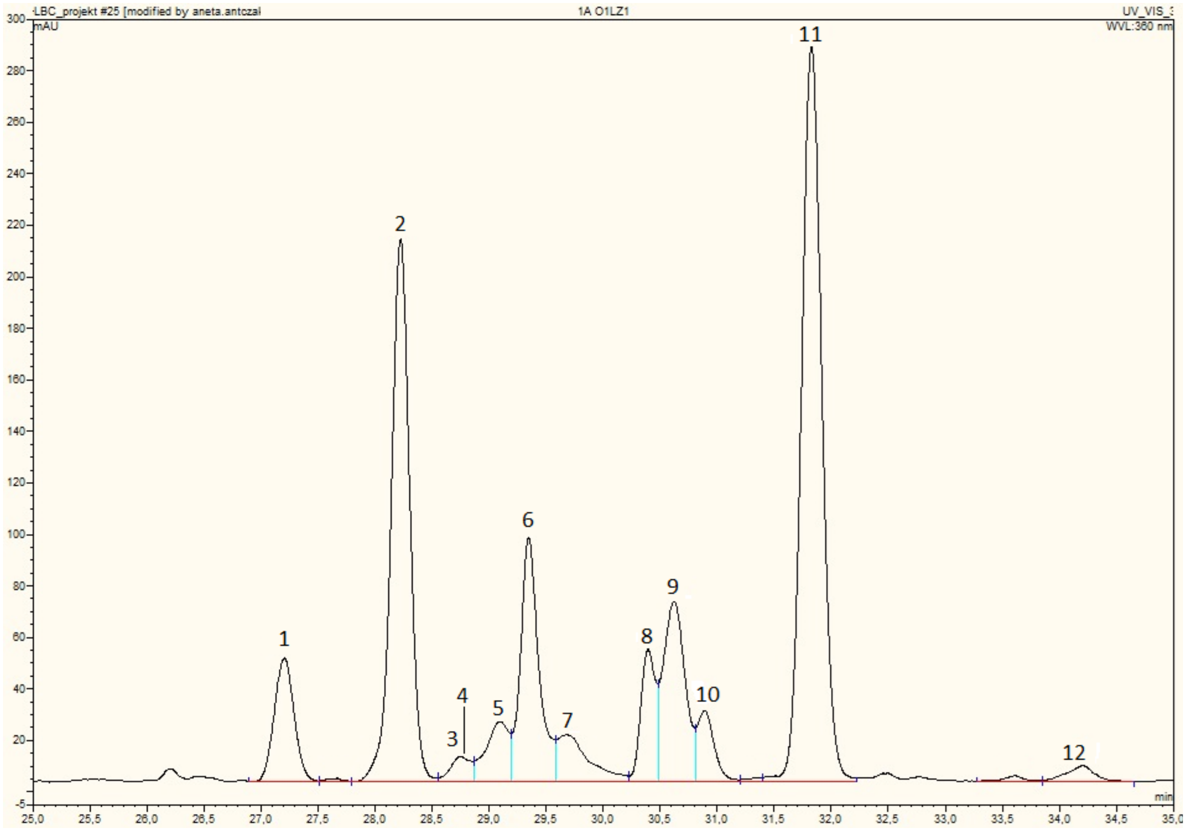

Figure S14. Example chromatogram HPLC-DAD of SBL sample

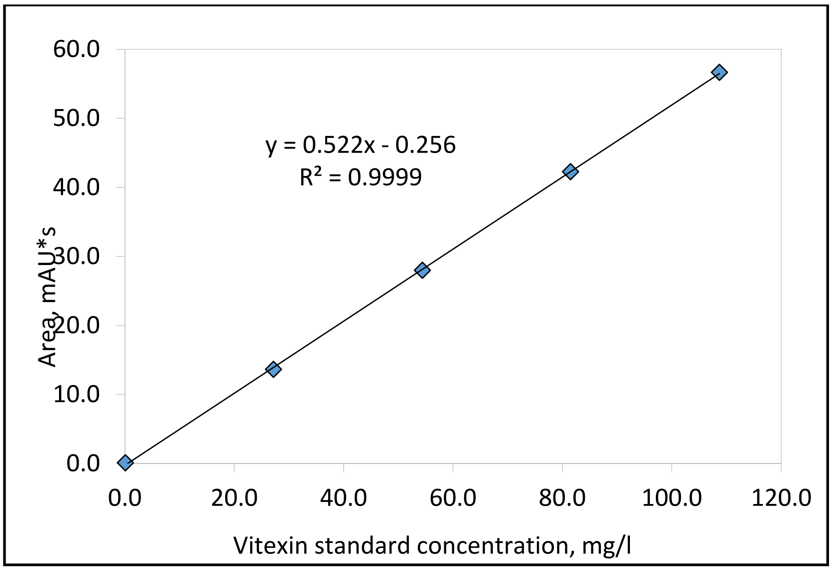

Figure S15. Calibration curve, linearity and coefficient of determination (R^2) of vitexin standard.

Table S1. Validation parameters for the determination of vitexin standard

	Vitexin
Linearity, mg/L	0.1 – 108.0
Calibration curve,	y = 0.522x – 0.256
Correlation ratio	R^2 = 0.9999
LOD, mg/L	0.02
LOQ, mg/L	0.06
Sr, µg/L	3.0
RSDr, %	2.8
Repeatability, µg /L	8.5
